# Job Satisfaction and Overcoming the Challenges of Teleworking in Times of COVID-19: A Pilot Study Among Iranian University Community

**DOI:** 10.1177/21582440231173654

**Published:** 2023-05-25

**Authors:** Fereshteh Ahmadi, Saeid Zandi, Mohammad Khodayarifard, Önver A. Cetrez, Sharareh Akhavan

**Affiliations:** 1 University of Gävle, Sweden; 2 Allameh Tabataba’i University, Tehran, Iran; 3 University of Tehran, Iran; 4 Uppsala University, Sweden

**Keywords:** coping strategies, occupational health, pandemic, telecommuting, working from home

## Abstract

The coronavirus pandemic changed the academic world in many ways, and most academic institutions continue operating through teleworking. The aim of the present study was to determine how satisfied the university community (faculty/staff members and students) in Iran has been with remote work, and the ways in which they have dealt with the lockdown and working from home during the coronavirus pandemic. A survey was conducted among 196 academics from different universities in Iran. The results show that a majority of our participants (54%) are very or somewhat satisfied with the current work-from-home arrangement. The most frequently used methods for managing the challenges of teleworking were *social contacts with colleagues or classmates at a distance*, *solidarity* and *offering kindness and support to the people around them*. The least used coping method was *trusting state or local health authorities in Iran*. The coping strategies that have the highest impact on overall teleworking satisfaction are “*Make myself busy with my working day because it makes me feel useful*,”“*I care for my mental and physical health*,” and “*Think about what I can do rather than what I can’t do*.” The findings were discussed in detail, taking into consideration the theoretical approaches, as well as bringing forth more dynamic aspects of the culture.

## Introduction

The outbreak of the coronavirus disease 2019 (COVID-19) in early 2020 changed the world in a variety of ways. Worldwide, the confirmed cases and deaths have grown rapidly and about 1,893,349 people have died due to COVID-19 at the time of submission of the present article (The Johns Hopkins University School of Medicine, 2021). The pandemic has been escalating, and this public health emergency may bring about a range of mental health aftereffects such as pandemic fatigue and also behavioral changes like stress eating and sleeping difficulties, and distress responses like depression and anxiety (N. Liu et al., 2020). The pandemic crisis has caused a condition that may lead to unfavorable effects on psychological and mental health among university staff and students (Al-Rabiaah et al., 2020; Araújo et al., 2020). Some research inquiries have investigated the impacts of the COVID-19 pandemic and self-isolation on students and staff in higher education. For instance, a cross-sectional study among 505 Bangladeshi college students showed that stress, depression and anxiety—from mild to extremely severe—had developed during the pandemic and lockdown. Further, worry about infection, financial uncertainties, and lack of physical and recreational activities were associated with depression, and anxiety symptoms (Khan et al., 2020). Another cross-sectional online survey among 2,530 staff and students of a Spanish University showed moderate to extremely severe scores on anxiety, depression, and stress among respondents. A total of 50.43% of participants reported a moderate to severe impact of the COVID-19 outbreak (Odriozola-González et al., 2020). Al-Nasa’h et al. (2021) conducted a study to estimate the students’ online learning satisfaction during COVID-19, showing that general anxiety and fear of COVID-19 were negatively associated with student satisfaction with distance education. Thus, COVID-19 has also influenced collegiate experiences and remote learning satisfaction. Most of the studies have argued that these adverse effects on academics are caused both by general COVID-19-related circumstances and the challenges of forced telework and stay-at-home orders (Ahmadi, Zandi, et al., 2022).

In Iran, on February 19th 2020, it was announced that two people in Qom city tested positive for coronavirus. Afterward, the disease spread to other provinces (Abdi, 2020). Iran ranked third in the number of confirmed cases after the People’s Republic of China and South Korea, and second in the mortality and recovery rate at the beginning of pandemic (Dong et al., 2020). At the time this paper was submitted, Iran still had high number of new cases and deaths. In January 6th 2021 the numbers were 6,283 new cases and 82 new deaths in Iran (Worldometer, 2021). For Iranians, as for people in many other nations in the world, this was the first experience of a health emergency with an imperceptible agent, resulting in high levels of uncertainty, and detrimental aftereffects for psychological health (Shigemura et al., 2020).

### Pandemic and Teleworking

COVID-19 epidemic has changed the working life of the workers, including their tasks, systems of supervision, and other work-related demands and activities (Chirombe et al., 2020). Therefore, one of the major effects of the ongoing epidemic was on workforce and occupational safety and health. The lockdowns and public health recommendations forced many people to work from home (Ahmadi et al., 2021; Lunn et al., 2020). Many organizations have adopted a “Work from Home (WFH)” approach to dealing with the crisis. Employees were forced to change their routine work practices and embrace other practices such as holding virtual meetings and flexible work arrangements. University community (here defined as faculty/staff members and students) also adopted this approach. They are facing a variety of challenges owing to the forced home-based telework following the outbreak of the pandemic. Most universities and colleges around the world have closed or canceled all campus events. Academic community has adjusted to online teaching platforms (Araújo et al., 2020; Sahu, 2020); however, students, faculty members, and academics’ attitudes, responses, reactions, and practices in the face of coronavirus varies to some extent from country to country.

In Iran, in response to the situation, between 29th February 2020 and 5th March 2020 there was progressive closure of schools and universities. Iran has more than 2,600 universities and colleges, which means that, during the pandemic, more than four million people have been working at home and based on forced adoption of distance education.

### Advantages and Challenges of Teleworking

Research has shown that the key advantages of working at home are increased autonomy and flexibility and that the disadvantages are possible sense of isolation and lack of separation between work and home (Harpaz, 2002). Hamblin (1995) has shown that the preferred option is working part of the week at home. A study from China showed that, during the COVID-19 pandemic, people who worked at their office had better mental health than those who worked at home. The study also demonstrated that those who worked at home reported more limitations regarding physical issues than did those who worked at offices (Zhang et al., 2020).

Pordelan et al. (2022) conducted a study among 404 Iranian working women in academic settings and showed that teleworking in the era of COVID-19 epidemic brings six major advantages for women, including economic, psychological, health, family, organizational, and educational advantages. However, their results demonstrated that some disadvantages (e.g., role conflict and lack of face-to-face position) are also attributable to telecommuting among academic teleworkers. In another Iranian study, Dastani (2021) examined different aspects of online education in Iranian medical universities during the COVID-19 pandemic. The findings of his study revealed that “the main challenges of online education in Iranian universities were college students’ non-equal accessibility to appropriate hardware, software, and communication tools, students and professors’ insufficient knowledge and unfamiliarity with information technology tools and e-learning, lack of proper interactions between professors and students, and the lack of a suitable platform for practical clinical training and internships.”

### Culture and Work Challenges

Since culture functions for a society in the same way as memory functions for a person, each culture provides culture-specific mechanisms for coping with stress and challenges (Kuo, 2011). Strategies for dealing with work stress and challenges that are appropriate in one context may be relatively ineffective in another (Pearlin & Schooler, 1978). Some research efforts show that culture may impact the way people choose to manage their work challenges. For example, Narayanan et al. (1999) compared coping strategies of American and Indian employees and demonstrated that Indians may have an external locus of control, in contrast to Americans’ more internal locus of control. Also, C. Liu et al. (2007), studying Chinese and North American university faculty, showed that American workers reported higher job autonomy as compared to Chinese workers, which they expected due to the greater levels of individualism in American culture compared to Chinese culture. Furthermore, O’Connor and Shimizu (2002), in their study, showed that Japanese college students were more likely to adopt emotion-focused coping strategies than British students.

As mentioned below, the current study is conducted in an Iranian academic setting; therefore, we here mention some characteristics of Iranian society. “Iran is a Middle Eastern country with a population of around 81,672,300 people (2018 estimate). Persian is the formal language in Iran. According to the Constitution of the Islamic Republic of Iran, the official religion of Iran is Shia Islam. Iran is quite well-known for its longstanding history and cultural heritage. Iranian people are proud of their rich literature and love Persian poets; poems of Hafez, Saadi, and Rumi are well-known also in western world for their mystical and moral ideas. Iranians usually read and sometimes memorize the poetry by these poets; some of these poems have turned into common expressions and proverbs among people in Iran” (Ahmadi, Khodayarifard et al., 2022, pp. 4–5). It is showed that this point also has significance for explaining our findings from a cultural perspective.

### Job Satisfaction and Teleworking

Job satisfaction or employee satisfaction is considered as a measure of workers’ contentedness with their job, whether they like the job or individual facets or aspects of job. Spector (1997) lists 14 common facets: “appreciation, communication, coworkers, fringe benefits, job conditions, nature of the work, organization, personal growth, policies and procedures, promotion opportunities, recognition, security, and supervision.”Locke (1976, p. 1304) defined job satisfaction as “a pleasurable or positive emotional state resulting from the appraisal of one’s job or job experiences.” According to Spector (1997, p. 39), “job satisfaction is assessed at both the global level (whether the individual is satisfied with the job overall), or at the facet level (whether the individual is satisfied with different aspects of the job).” In the current study, by job satisfaction we mean the global level (overall job satisfaction).

L. Johnson (2016) found that job satisfaction among telecommuters was high. It is argued that the fact that telecommuting employees can save the commute times and the gas expenses or transportation costs may increase their satisfaction with remote work. Dubrin (1991) showed that the individuals who worked in a work-at-home arrangement experienced higher job satisfaction as compared to in-house (i.e., office-based) employees. In that study, the scheduling of own working hours and the opportunity to take care of family and personal responsibilities have been introduced as the key factors in increasing teleworkers’ satisfaction.

Some similar studies have been recently conducted during the current pandemic. For example, F. Tavares et al. (2021) found that adjusting to working from home was easy or very easy and that it turned to happen very quickly in Portuguese communities during the coronavirus pandemic. Arora and Vyas (2020) demonstrated that most remote workers are satisfied with their jobs at times of COVID-19. Baert et al. (2020) investigated perceptions of telework during the COVID-19 crisis among Flemish employees and found that 65.7% of respondents indicated that their satisfaction with their job had increased while working from home and that the respondents mainly attributed positive characteristics to teleworking, for example, lower risk of burnout and increased efficiency.

Although several studies have claimed that job satisfaction is higher among telecommuting people, the findings of some other studies have been inconsistent with this claim (Bailey & Kurland, 2002; Morganson et al., 2010). Golden and Veiga (2005) examined the impact of the intensity of remote work on the job satisfaction of teleworkers as an attempt to resolve the inconsistent findings. They demonstrated that there is a curvilinear relationship between the intensity of telecommuting and employee satisfaction, with job satisfaction appearing to plateau at higher levels of telecommuting (Barbieri et al., 2021). Moreover, telecommuting was positively associated with employee satisfaction for employees who teleworked for less than 15.1 hr a week. This positive correlation disappeared when the employee teleworked more than 15.1 hr. Golden and Veiga (2005, p. 303) hypothesized that, for high-intensity remote workers, “the negative impact of increased isolation and decreased social interaction on relationships with supervisors and coworkers is likely to negatively affect job satisfaction.”

### Aim of This Study and the Research Questions

As mentioned, the level of teleworkers’ job satisfaction may be different from that of office workers. However, little research has been done to determine how satisfied an individual, who has not worked from home so far, is with an involuntary work-from-home arrangement. Specifically, to date, and to the authors’ knowledge, there is a paucity of data on satisfaction with working from home among Iranian university community. Also, the methods the Iranian university community members use to overcome the challenges of forced teleworking seem to be interesting and notable. Thus, the aim of this study is to determine how satisfied our sample of Iranian university community (faculty/staff members and students) has been with remote work, and the ways in which they have dealt with the lockdown and working from home during the coronavirus pandemic. The following research questions are addressed:

**Question 1 (Q1):** To what extent is our sample of Iranian university community satisfied with the work-from-home arrangement during the ongoing pandemic?
**Question 2 (Q2):** What are the ways in which our sample of Iranian academic community has tried to manage the challenges of working from home during the coronavirus pandemic?

To gain a theoretical understanding regarding our aims and questions, we believe in the value of using Job Demand-Control (-Support) model.

### Job Demand-Control (-Support) Model

Job Demand-Control model (Karasek, 1979), also known as the job strain model, is one of the theoretical models explaining the factors that lead to job satisfaction and coping with the challenges of working. This model provides an explanation for labor stress in terms of the balance between the job’s demands and the extent to which the worker has control over these. “Job demands” refer to the work load (e.g., role conflict, time pressure, etc.). “Job control,” also called decision latitude, refers to the individual’s ability to control her/his occupational activities. The worker’s health and satisfaction depend on the balance between the job demands and the worker’s resources. According to Besen (2013, p. 24),

at high levels of job demands, individuals experience a state of arousal, or stress, characterized by increased adrenalin levels. … According to the Job Demands-Control model, in jobs with high levels of control, the state of arousal can be counterbalanced through coping mechanisms (i.e., control), but in jobs with high demands and low levels of control, the state of arousal remains, ultimately leading to poor health outcomes.

Stress-like reactions result when there are restricted opportunities for action or coping with the stressor. Person-based coping strategies and feelings of mastery or confidence can lead to reduced perceptions of events as stressful and increased satisfaction. Later, a social dimension was also added to this theoretical model (J. V. Johnson & Hall, 1988; J. V. Johnson et al., 1989), resulting in the Job Demand-Control-Support (JDCS) model. “The social dimension (i.e., Job Support) refers to the extent to which the employee gain emotional support, such as someone to talk to, instrumental support, such as getting help with a task, informational support, such as getting work-related information, and appraisal support, such as feedback about one’s performance” (Besen, 2013, p. 15). In the expanded version of the model, “job demands, job control and worksite social integration are crucial aspects in the development of health problems” (van der Doef & Maes, 1999, p. 89). Therefore, according to JDCS model, the employees can decrease the challenges and work-related stress through two resources: (1). Developing strong relationships with supervisor and colleagues (2). Gaining greater job control. We should note that when work is done remotely from home, gaining job control is better facilitated thorough coping strategies than social support, as compared to on-site work in which a variety of supports are more likely available. According to Tietze (2002, p. 388),

in the homeworking literature, the need to find practical solutions to teleworking is generally acknowledged and captured by the term coping strategy. Coping strategies address the redrawing of (cultural) boundaries around “work” and “home” in the absence of physical distance. … Coping strategies address the practicalities of accommodating the co-presence of “work” and “home”.

The JDC(S) model has been applied in some studies conducted during the ongoing global pandemic (Norful et al., 2021; Shacham et al., 2020). Due to changes in the workplaces and work-related tasks during the current epidemic, it is expected that employees have experienced changes in all three dimensions of JDCS model. Therefore, their health and job satisfaction will depend on the balance between the job demands in the new work-from-home environment and the worker’s available resources. Accordingly, social support, decision-making freedom, and coping strategies can be considered crucial resources to managing the challenges during remote work in the era of COVID-19. It is worth noting that as this is an unprecedented situation, there are almost no previous studies that have focused on mandatory full-time telework. Furthermore, as Barbieri et al. (2021) argued, working from home full-time implies new and different demands and resources. Although several resources could potentially contribute to the health and satisfaction of employees during the current pandemic, we had to determine some condition-adjusted coping resources for this study. Therefore, given the peculiarities of teleworking and the novelty of the COVID-19 situations, and also due to the urgency of investigating the topic and the potential applications and implications of the study findings, we listed potential coping strategies adjusted to a mandatory work-from-home condition during COVID-19-related lockdowns and based on the theoretical model’s components Control and Support (for more information on coping strategies examined in this study, please see Appendix). In addition, since the university community are unlikely to develop and maintain strong relationships with colleagues and classmates during working from home as compared to on-campus face-to-face interactions, the social support dimension (i.e., informational support, emotional support, appraisal support, and instrumental support) may have limited impact on their wellbeing and satisfaction. Therefore, it is likely that academic community benefits more from the Job Control dimension in their attempts to manage the challenges of teleworking.

## Methodology

A quantitative research design was used to conduct this cross-sectional study. The variables were as follows: job satisfaction, coping strategies, self-reported health, place of residence, age group, gender, and work/student status.

### Sample Characteristics

The target group for this study was academic community (faculty/staff members and college students) studying/working at different universities in Iran. We used an available list to invite the potential participants. This approach was found most convenient since for the target group email addresses were available (Fricker, 2008). The sample size was 196. Table 1 demonstrates the demographic characteristics of the participants. A clear majority of respondents are female. Almost 61% are students while 39% are staff/faculty members. The large majority, 56%, are single, 38% are married, and very few are divorced or engaged. Seventy-seven percent of respondents do not have any children. Finally, the majority of respondents live in the capital. Therefore, our sample skewed female, younger, single, and student respondents. This sample was also used in a previous publication by Ahmadi et al. (2023) which reported the psychological resilience of the academic population during the mass trauma of the current global pandemic.

**Table 1.** Respondents’ Demographic Characteristics (*n* = 196).

Variable	Frequency (%)
Gender	
Male	49 (25)
Female	147 (75)
Age (years)	
<25	61 (31)
25–35	76 (39)
>35	59 (30)
Education	
High school or similar	4 (2)
University	192 (98)
Country of birth	
Iran	194 (99)
Afghanistan	2 (1)
Country of residence	
Iran	194 (99)
Switzerland	2 (1)
Work/student status	
Full-time employment	49 (25)
Part-time employment	27 (14)
Campus student	43 (22
Distance learning student	77 (39
Civil status	
Married	74 (38)
Divorced	4 (2)
Engaged	8 (4)
Single	110 (56)
Children	
Children	45 (23)
No children	151 (77)
Place of residence	
Capital	114 (58)
Medium–large city	33 (17)
Small town, close to a large city	37 (19)
Small town, far from a large city	12 (6)

*Source*. Reprinted from “Living through a Global Pandemic: A Cross-Sectional Study on the Psychological Resilience of the University Population in Iran,” by Ahmadi et al. (2023).

### Data

The data were gathered using Cafepardazesh, which is an Iranian online survey maker. The link to the online questionnaire was e-mailed to potential informants. In the email, there was an invitation letter explaining the study and asking the recipients to voluntarily take part in the study. As mentioned before, the participants were recruited from an existing list. The list comprised of 885 academic community members and the invitation email was sent to all persons on the list on 30 May 2020. A total of 210 respondents returned the questionnaire until 9 June 2020, when the data collection was terminated. Given the missing data, 196 questionnaires were included in the data analysis. It should be mentioned that we have not a random sample and we do not aim to generalize our findings to the whole population. We have presented the results concerning only our sample.

### Measure

In this study and our other concurrent investigations, we did not make use of the existing questionnaires due to the novelty of the pandemic situations. Given the urgency of investigating the topic and the potential implications of the results, we constructed a questionnaire including items adjusted to a homebound working condition during COVID-19. The questionnaire included four main questions (see Appendix): The first question asked whether the individual was working more than the contracted hours when working from home (responses, *yes*/*no*). The second question asked how satisfied the person was with working from home (responses ranging from 1—*Very dissatisfied* to 5—*Very satisfied*); it should be mentioned here that since university students generally do a variety of academic affairs and activities (e.g., studying and learning, participating in classrooms, group work assignments, presenting lectures at online classrooms, researching, writing reports, serving as research/teaching assistant etc.), this question captures the students’ overall satisfaction with remote academic working, and not merely their satisfaction with distance learning; also, it should be noted that since the aim was to assess the overall job satisfaction as defined by Spector (1997), we used only one item to capture the academics’ satisfaction with working from home. The third question asked how the individual was coping with the challenges of teleworking, and included 12 sub-questions (responses ranging from 1—*Never* to 5—*Always*) with a Cronbach’s alpha of .748, which is considered acceptable (George & Mallery, 2016). In addition, a fourth main question about general self-reported health was asked (responses ranging from 1—*Poor* to—*Excellent*). It should be noted that three experts with doctoral degree in behavioral and social sciences reviewed and finally confirmed the validity of the variables. This instrument has been also validated for form and content in the concurrent studies conducted by the research team (e.g., Ahmadi, Zandi, et al., 2022).

### Data Analysis

Since a convenience sampling method was used, representativeness and generalizability of the findings are restricted; therefore, we have not conducted any calculations of statistical significance. To analyze the data, we performed cross tabulations (by age group [young, middle, older], gender [female and male], place of residence [capital, medium-large city, small town close to a large city, small town far from a large city], and work/student status [full-time, part-time, on-campus student, distance-learning student]); it should be noted that the results of these crosstabs are presented via charts. We also performed factor analysis, Pearson’s correlation, and linear regression tests. The regression has five key assumptions which the analysis met up to: (1) Linear relationship, (2) Multivariate normality, (3) No or little multicollinearity, (4) No auto-correlation, (5) Homoscedasticity. The factor analysis has five assumptions which were met: (1) Correlations are linear, (2) No outliers, (3) No multicollinearity, (4) Reasonably high correlations are present (5) Moderately skewed distributions. Statistical analyses were conducted using Microsoft Excel and IBM SPSS Statistics 27.

### Ethical Considerations

In a letter appended to the questionnaire, the participants were informed about the research project, their ability to withdraw, and the use and preservation of the data. The participants were also informed that responding to the questionnaire would be considered as giving consent. The Swedish Ethical Review Authority approved the study for the parts linked to Sweden: data analysis and data preservation (Reg. no. 2020/ 02368 9). For the present study, an internal group at the University of Tehran also checked the research proposal and questionnaire and approved them.

## Results

It is notable that since the size of sample was limited and also it skewed female, younger, single, and student respondents, we cannot maintain that our sample is representative of the totality of the teleworking academics in Iran, and the obtained results regard only the respondents in this study. Our results cannot therefore be generalized to all academic community in Iran.

### Self-Reported Health

**Figure 1.**
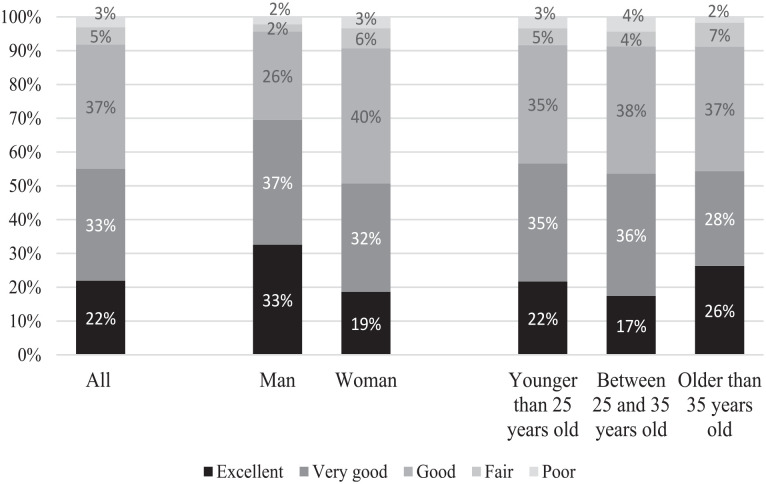
Participants’ self-reported health, by gender and age. *Source*. Adapted from “Living through a Global Pandemic: A Cross-Sectional Study on the Psychological Resilience of the University Population in Iran,” by Ahmadi et al. (2023).

Figure 1 presents self-reported health among academics from Iran, who perceive that they are quite healthy. Overall, 55% report their health is excellent or very good. Another 37% report that their health as good. This means that 92% report at least good health. Only 3% report ill health. Men more often report their health is excellent (33%) or very good (37%); the corresponding figures for women are 19% and 32%. Across ages there are only very small differences, as 57% of the young respondents report their health is excellent or very good, and the corresponding figure is 53% for those 25 to 35 years of age and 54% for those older than 35 years. It is notable that the older group more often say that their health is excellent.

### Teleworking and Job Satisfaction During COVID-19

**Figure 2.**
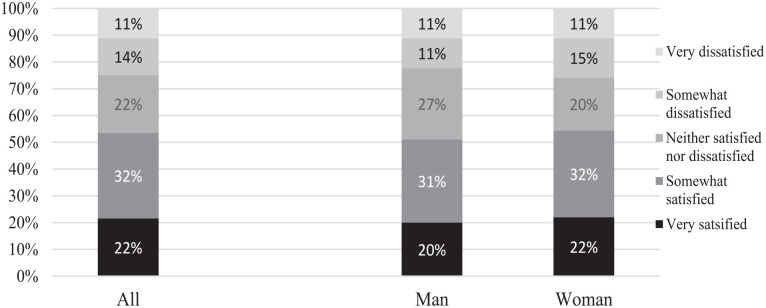
Level of satisfaction with teleworking, by gender.

The findings show that 4 in 10 academics in Iran perceive that they work more than contracted hours after having started to work from home due to the COVID-19 crisis. Figure 2 presents the level of satisfaction with teleworking, where a majority (54%) is very or somewhat satisfied with the current work-from-home arrangement. These results are very even between men and women, although men more often than women perceive that they are neither satisfied nor dissatisfied with teleworking (Figure 2).

### Coping Strategies Employed to Manage the Challenges of Work-From-Home

**Figure 3.**
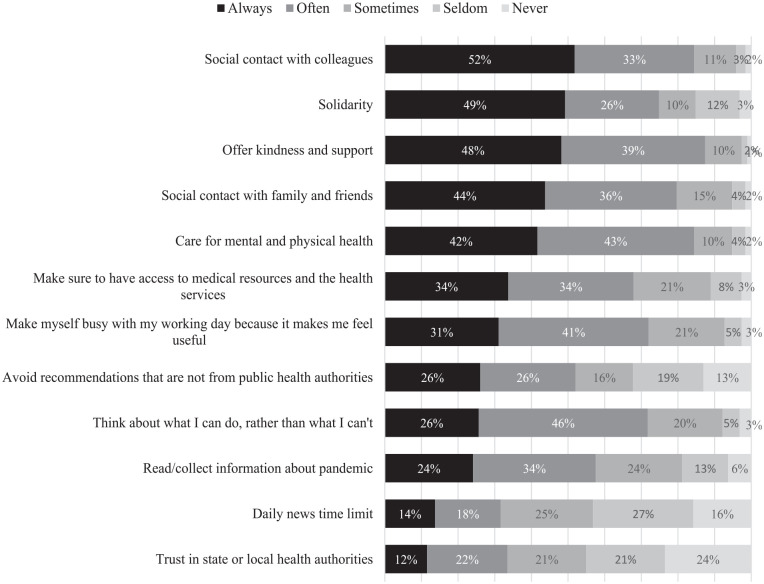
Coping strategies employed to manage the challenges of work-from-home.

More than 90% of the participants use nine of 12 coping methods to deal with the challenges of teleworking during the COVID-19 pandemic. Figure 3 shows 13% never use “avoiding recommendations that are not from public health authorities in my country or from the World Health Organization” (*M* = 3.33, *SD* = 1.38), 16% never use “giving themselves a time limit for daily consumption of news” (*M* = 2.86, *SD* = 1.27) and 24% never use “trusting state or local health authorities in my country” (*M* = 2.77, *SD* = 1.33).

Figure 3 is ranked after coping methods used to deal with the challenges of teleworking. A majority of respondents always have “social contacts with colleagues or classmates at a distance” (*M* = 4.30, *SD* = 0.88). Almost half report “they are in this together and through solidarity they find the best solutions” (*M* = 4.06, *SD* = 1.16). “Trying to offer kindness and support to people around them” (*M* = 4.32, *SD* = 0.80) was another important way to cope with the pandemic and challenges of working from home. Additionally, more than four in 10 always have social contact with friends and family through social media or distance tools (*M* = 4.16, *SD* = 0.92), or always care for their mental and physical health (*M* = 4.20, *SD* = 0.88). Table 2 shows the descriptive statistics of the coping methods in detail.

**Table 2.** Descriptive Statistics of the Coping Methods Used by Participants.

	I have social contact with my colleagues/classmates through distance tools and other social media	I have social contact with my family and friends through distance tools and social media	I think about what I can do, rather than what I can’t	I make myself busy with my working day because it makes me feel useful	I read/collect information from Public health authorities in my country or World Health Organization and keep myself update with public health news	I trust state or local health authorities in my country	I give myself a news time limit for each day	I avoid recommendations that are not from public health authorities in my county or from World Health Organization.	I care for my mental and physical health	I try offering kindness and support to the people around me	I make sure to have access to medical resources and the health services if I need to seek health care	I believe we are all in this together, and with solidarity we can find the best solutions for handling covid-19
*N*	195	193	192	195	192	192	191	193	193	192	191	192
Mean	4.30	4.16	3.86	3.93	3.56	2.77	2.86	3.33	4.20	4.32	3.87	4.06
Median	5.00	4.00	4.00	4.00	4.00	3.00	3.00	4.00	4.00	4.00	4.00	4.00
Mode	5	5	4	4	4	1	2	4^a^	4	5	4	5
Std. Deviation	0.888	0.924	0.958	0.976	1.165	1.335	1.270	1.381	0.880	0.799	1.054	1.165
Variance	0.789	0.854	0.917	0.952	1.357	1.782	1.613	1.909	0.774	0.639	1.110	1.357
Skewness	−1.392	−1.085	−0.907	−0.843	−0.534	0.131	0.196	−0.305	−1.278	−1.333	−0.728	−1.066
Range	4	4	4	4	4	4	4	4	4	4	4	4
Minimum	1	1	1	1	1	1	1	1	1	1	1	1
Maximum	5	5	5	5	5	5	5	5	5	5	5	5

*Note*. The items are reproduced from the original scale by Ahmadi, Zandi, et al. (2022).

a Multiple modes exist. The smallest value is shown.

To better understand these 12 strategies for coping with the challenges of telecommuting at times of COVID-19, an automated factor analysis was performed. It is worth noting that with factor analysis we can, by watching the relationship between different variables, find possible underlying factors instead of analyzing the single variables. The purpose of factor analysis is simply to find these latent, not observed variables, from the observed variables. Let’s see in a simple way how an exploratory factor analysis can be performed, which is one of the most used in social sciences. It should be noted that the points mentioned below can be selected in statistical programs such as SPSS when performing the analysis.

- **Reliability analysis:** Normally, Cronbach’s Alpha is used, which allows one to know the internal consistency of the model. Values greater than 0.70 are considered acceptable.
- **Descriptive statistics:** These provide us with basic information about the data analyzed. The mean, the variance or the maximum and minimum.
- **Correlation matrix analysis:** These calculations are performed by SPSS. Here we have to pay attention to whether the determinant is close to zero. On the other hand, the calculated correlations must differ from zero.
- **KMO sample adequacy:** Allows us to contrast the correlation coefficients. On the one hand the observed and on the other the partial. It takes values between 0 and 1 and is considered acceptable if greater than 0.5.
- **Bartlett’s test of sphericity:** In this case, in contrast, the correlation matrix is an identity matrix, in which case the analysis could not be done. The estimated Chi-square is calculated and if it is less than the theoretical one, the actual analysis can be done.
- **Analysis of commonality:** Again, it is an indicator of relevance. To be valid, it must take values greater than 0.5.
- **Rotated component matrix:** It is used to extract the eigenvalues that are greater than a value, normally 1. In this way, the reduced factors that represent the variables are obtained. Sedimentation charts and the matrix itself are used to select numbers.
- **Total variance explained:** Finally, this analysis tells us what is the total variance explained by the proposed model. The higher this value is, the better the model is at explaining the total data set.

In this study, SPSS factor analysis has been used with the method of Varimax rotated solution. In the analysis, all the coping methods in the survey were included. When one single variable does not correspond to any other variables from the questionnaire it creates a factor based on only one variable. And sometimes a factor only consists of two variables. This means that some variables, or statements in the questionnaire are unique in this battery of statements since it has an own underlying factor. If more variables were added to the battery of coping methods the factor would then probably be the underlying factor for more variables. Here, the factor analysis is based on the surveyed data only, and the result from this factor analysis represents merely this sample.

In this case, four factors were generated. Four of the 12 coping strategies were often mentioned together, yielding a factor called “Being active factor.” Another three coping methods were grouped to create another factor, “Trust and information factor.” A third way of addressing the situation was “Medical and solidarity factor.” The fourth factor was named “Social factor.” The item “I avoid recommendations that are not from public health authorities in my county or from World Health Organization” is removed since it was not part of any factor (See Table 3; note that the patterns in the table show which coping methods belong to which factor).

**Table 3.** Factor Analysis of Different Ways for Coping With the Challenges of Telecommuting.

	Component			
	Being active factor	Trust and information factor	Medical and solidarity factor	Social factor
I think about what I can do, rather than what I can’t	0.748	−0.016	0.064	0.104
I make myself busy with my working day because it makes me feel useful	0.621	0.159	−0.013	0.107
I care for my mental and physical health	0.717	0.148	0.261	0.167
I try offering kindness and support to the people around me	0.782	0.046	0.140	0.129
I read/collect information from Public health authorities in my country or World Health Organization and keep myself update with public health news	0.150	0.808	0.094	0.146
I give myself a news time limit for each day	0.078	0.866	−0.006	0.085
I trust state or local health authorities in my country	0.010	0.622	0.524	−0.080
I make sure to have access to medical resources and the health services if I need to seek health care	0.032	0.056	0.826	0.339
I believe we are all in this together, and with solidarity we can find the best solutions for handling COVID-19	0.334	0.110	0.755	0.055
I have social contact with my colleagues/classmates through distance tools and other social media	0.164	0.083	0.154	0.834
I have social contact with my family and friends through distance tools and social media	0.208	0.104	0.129	0.849

*Note*. The items are reproduced from the original scale by Ahmadi, Zandi, et al. (2022). The colors are only to indicate which items are included in each factor.

### Coping Strategies, by Age and Gender

**Figure 4.**
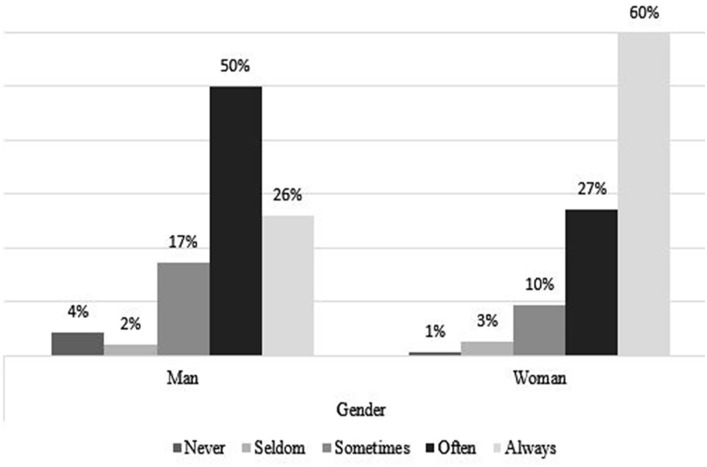
Social contact as one of the most frequently used methods for coping with the challenges of teleworking by gender.

**Figure 5.**
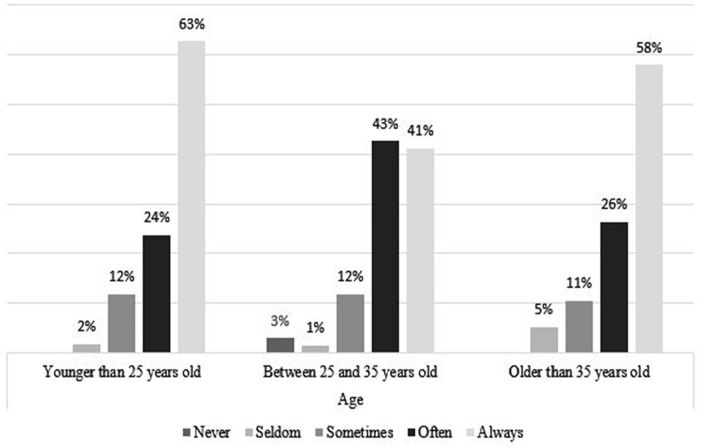
Social contact as one of the most frequently used methods for coping with the challenges of teleworking by age.

**Figure 6.**
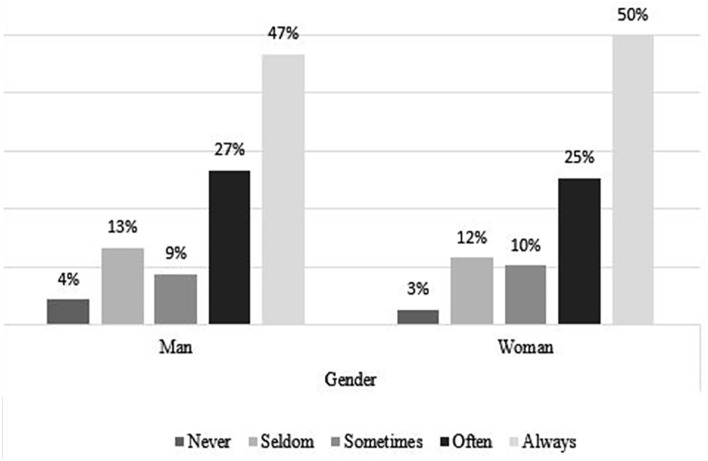
Solidarity as one of the most frequently used methods for coping with the challenges of teleworking by gender.

**Figure 7.**
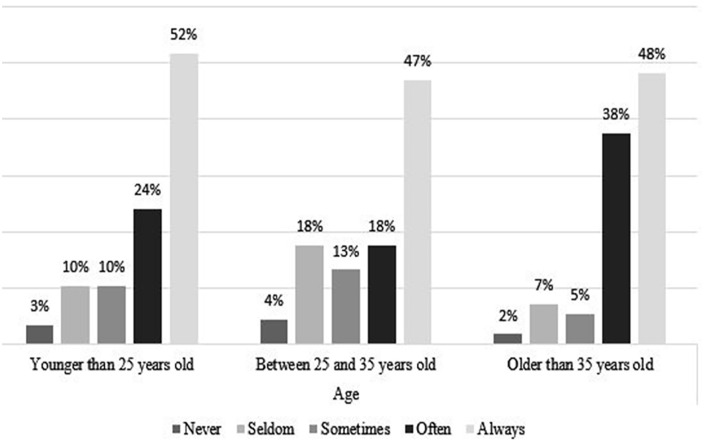
Solidarity as one of the most frequently used methods for coping with the challenges of teleworking by age.

**Figure 8.**
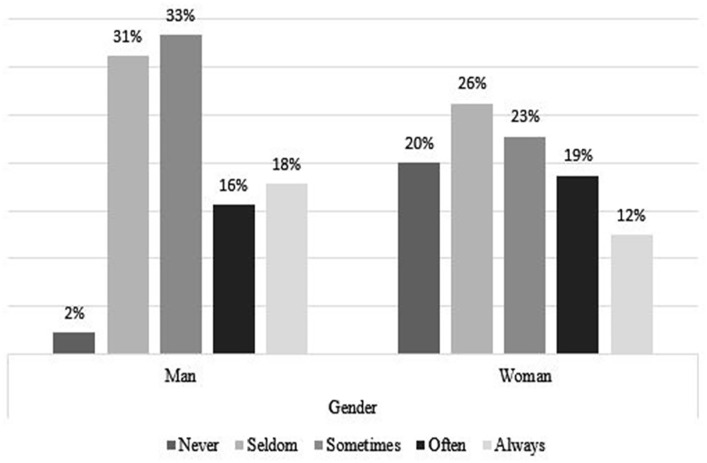
Using daily time limits as one of the least frequently used methods for coping with the challenges of teleworking by gender.

**Figure 9.**
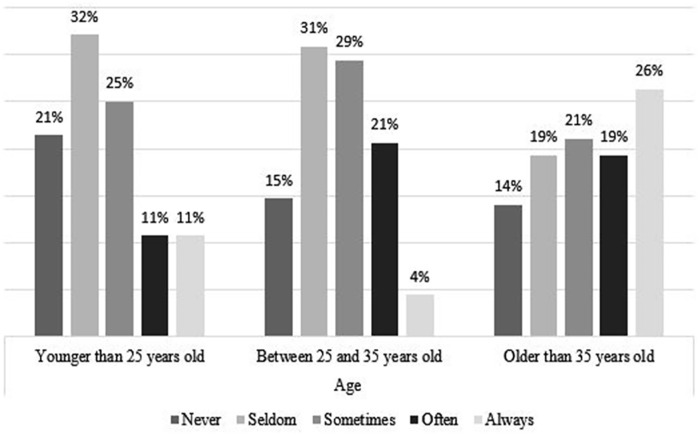
Using daily time limits as one of the least frequently used methods for coping with the challenges of teleworking by age.

**Figure 10.**
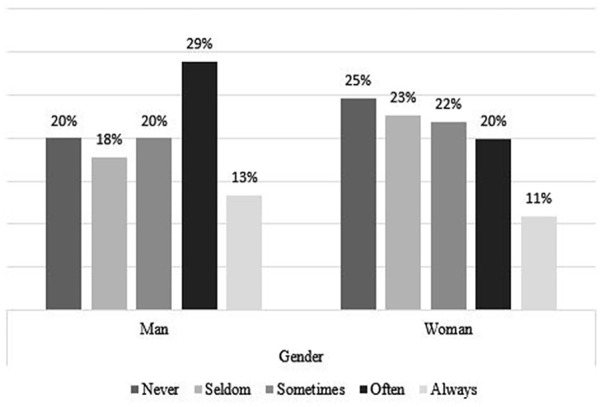
Trust in state or local health authorities as one of the least frequently used methods for coping with the challenges of teleworking by gender.

**Figure 11.**
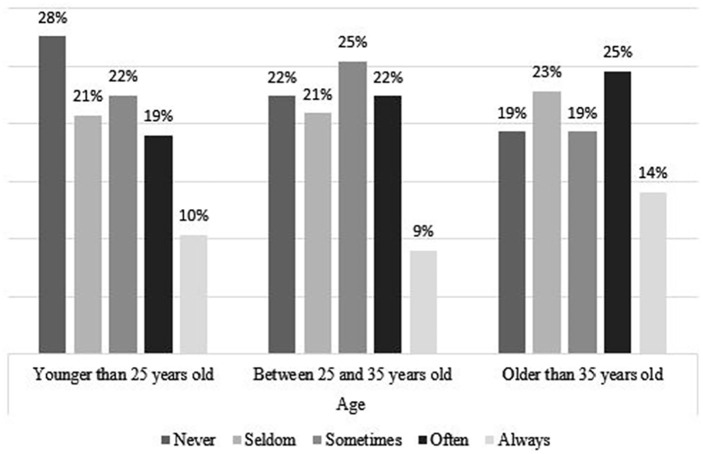
Trust in state or local health authorities as one of the least frequently used methods for coping with the challenges of teleworking by age.

Figures 4 to 11 show the most and least frequently used coping methods for dealing with the challenges of teleworking by gender and age. In these figures, use of a coping method refers to the response values *Always*, *Often*, *Sometimes*, *Seldom*, and *Never*. Figure 4 shows that women use social contact with colleagues/classmates through distance tools and other social media to deal with the challenges of teleworking more than men do. Respondents younger than 25 years of age, more than the other age groups, report using social contact with colleagues/classmates through distance tools and other social media to deal with the challenges of teleworking (Figure 5). Solidarity, as a method of coping with the challenges of teleworking, is also used by more than 40% in all ages and of both sexes (Figures 6 and 7). Limiting the daily time spent on news consumption is used most by those 35 years and older as a method for coping with the challenges of teleworking (Figure 9). Among all respondents, the young group are the least likely to use trust in state or local health authorities as a method of coping with the challenges of teleworking (Figure 11).

### The Impact of Coping Strategies on Teleworking Satisfaction

**Figure 12.**
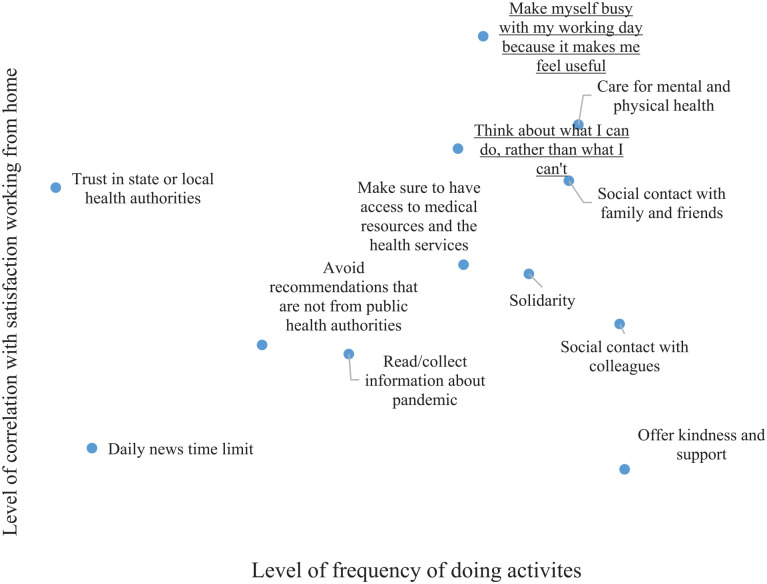
Relative impact of coping strategies on teleworking satisfaction.

The coping methods that have been used often are not always the most important ones. The coping strategies that have the highest impact on overall job satisfaction during pandemic are shown in Figure 12. When all of the coping strategies are correlated with overall job satisfaction, it is seen that doing activities and keeping busy seem to have the greatest positive impact on teleworking satisfaction, as the coping methods “Make myself busy with my working day because it makes me feel useful” (*M* = 3.93, *SD* = 0.97) and “Think about what I can do rather than what I can’t do” (*M* = 3.86, *SD* = 0.95) are illustrated. Also, the coping strategy “I care for my mental and physical health” is among the methods with greatest positive impact on job satisfaction. The values of linear regression and correlation tests are shown in Table 4.

**Table 4.** Impact of Coping Strategies on Teleworking Satisfaction.

	Mean based on frequency	Regression (*b*-value)	Correlation
Social contact with colleagues	4.31	−0.023	.09
Social contact with family and friends	4.17	0.151	.16
Think about what I can do, rather than what I can’t	3.86	0.189	.25
Make myself busy with my working day because it makes me feel useful	3.93	0.325	.34
Read/collect information about pandemic	3.57	−0.059	.05
Trust in state or local health authorities	2.76	0.142	.12
Daily news time limit	2.86	−0.173	.00
Avoid recommendations that are not from public health authorities	3.33	−0.048	.05
Care for mental and physical health	4.19	0.218	.18
Offer kindness and support	4.32	−0.198	.09
Make sure to have access to medical resources and the health services	3.88	0.049	.16
Solidarity	4.06	0.038	.16

## Discussion

The present research investigated satisfaction with teleworking during the COVID-19 pandemic among a sample of Iranian academics. The study also made attempts to explore the ways in which the participants cope with the challenges of pandemic-related lockdown and working from home. The majority of participants were healthy, single, young, female university students who live in the capital city of Iran. It should be noted that the obtained findings regard the respondents in this study. We have no ambitions to generalize our findings to the whole population.

### Government-Mandated Teleworking and Job Satisfaction

The results show that a majority of respondents (54%) are very or somewhat satisfied with the current work-from-home arrangement (Q1). This finding is consistent with F. Tavares et al. (2021) and Arora and Vyas (2020). The positive effect of teleworking on job satisfaction is also in line with Baert et al. (2020). It is worth noting, however, that some studies (e.g., Raišienė et al., 2020) have compared perceptions of telework between individuals who began working from home for the first time during the COVID-19 quarantine period and those with longer telework experience, showing that those only teleworking during the quarantine (who formerly worked in a physically common workspace) tended to emphasize the drawbacks of telework less. Therefore, it seems that some longitudinal studies are needed to decisively talk about job satisfaction with the relatively temporary period of teleworking during health pandemics. In addition, academics, given their working conditions, are unlikely, on average, to telework very long hours a week. Therefore, they may not be considered “high-intensity remote workers,” in the words of Golden and Veiga (2005), to be negatively affected by telework to a great extent.

Job satisfaction in a teleworking context during the COVID-19 pandemic may be attributed to the flexibility one has in timing, choosing workhours, and clothing as well as the ability to save time normally spent on commuting. Research has shown that teleworking provides staff with flexibility and autonomy (Harpaz, 2002). Autonomy has often been correlated with telecommuting as working away from direct supervision, people have higher levels of autonomy to organize, plan, and execute work-related activities (Standen et al., 1999). Teleworkers, as compared to office workers, more easily can choose to execute their work-related roles and duties in a way that is right for them (Brunelle & Fortin, 2021). Additionally, teleworking allows work to be combined with household chores, and it ultimately improves the balance between work and family (A. I. Tavares, 2017). Karasek’s job demand-control-support model (Karasek, 1979) argues that the experience of work stress and dissatisfaction is a consequence of the imbalance between job demands and the individual’s perception of their social support and job control. According to the theoretical framework we proceeded from, working from home, our participants may have gained increased freedom in making decisions concerning the ways in which to work. This may have contributed to balance between the job demands and individuals’ available resources, resulting in relative satisfaction with home-based working.

Because the majority of respondents in our sample were women and also women’s job satisfaction was slightly higher than men’s, we may also think about possible gender effects on teleworking satisfaction. The traditionally-expected gender roles, apparently assigning woman more responsibilities in her daily life than man is assigned, may provide an explanation. Specially, this may be the case in our study as our cohort has been selected from an Islamic country, in which women, whether single or married, are more likely expected to be more involved in household, and family care activities. Therefore, it seems to be more difficult for women to combine job-related responsibilities with their personal responsibilities. Telework may be a way to facilitate this combination; this hypothesis has also been presented by Baert et al. (2020). Raišienė et al. (2020), in their comprehensive research, also found that women particularly appreciate the opportunity to telework from home to ensure a healthier lifestyle during the COVID-19 lockdown.

### Coping Strategies Employed to Face the Challenges of Work-From-Home

The present findings demonstrated that the most frequently used strategies for dealing with the challenges of telecommuting during the pandemic-related lockdown are “social contacts with colleagues or classmates at a distance,”“solidarity,” and “offering kindness and support to people around them” (Q2). Having social contacts at a distance may be considered a substitute for typical face-to-face relationships and in-person activities, and it may bridge the gap of loneliness during the imposed social isolation restrictions, where individuals are confined to their homes, thus enhancing homebound people’s ability to better cope with the challenges and worries. In their studies, Emerson (2020) and Moore and March (2020) have also demonstrated that use of media connections (phoning, texting, Facebook, and Instagram) to liaise with others would help people to cope better with the challenges of the COVID-19 situation and forced social distancing and home confinement derived from the pandemic.

Solidarity and offering support to others—the second and third most frequently used coping methods by our sample of Iranian academics during the current global health crisis—have also been found in other studies. For example, Jenkins (2020) showed that facing the challenges of COVID-19 is facilitated through solidarity, compassion and empathy. These coping strategies are reminiscent of a pattern of altruism. Ahmadi (2006, p. 139) explained: “Human altruism is not only an act, but is interwoven with an emotion: empathy. Altruism is then a prosocial behavior, a voluntary intentional act to help others at some cost to oneself (time, effort or money).”Ahmadi, Khodayarifard et al. (2022), who studied an Iranian sample, showed that these coping methods are widely used in times of crisis and illness. Moreover, Hartman and Morse (2020) found that a crisis usually causes solidarity and that experiences of collective trauma and hardship increase empathetic concern. They argue that “empathetic concern transcends identity boundaries and motivates altruistic behavior” (p. 737). Altogether, effective responses to the worries caused by the COVID-19 pandemic may be reinforced via positive practices like empathy and solidarity. We have all witnessed all of the truly human things that have been happening around the world, such as balcony singing in Italy and the people sewing masks where there are supply shortages. The least used coping method found in our study is “trusting state or local health authorities in Iran.” This is in line with the Gallup Poll (2020), which recently reported that the Iranian people’s confidence in their government was under 50% for the first time. This may be explained by the economic crisis as well as the fact that the country has become the COVID-19 epicenter in the Middle East.

The present findings also demonstrated that the coping strategies with the highest impact on overall teleworking satisfaction when a pandemic strikes are “Make myself busy with my working day because it makes me feel useful,”“I care for my mental and physical health,” and “Think about what I can do rather than what I can’t do.” According to the conceptual model we have proceeded from (i.e., JDCS model), these coping strategies are related to personal resources than social resources. This result might be explained by attending the fact that our respondents have not had access to supportive in-person relationships with their colleagues and classmates during working from home; this is, interestingly, despite the fact that they have resorted to the coping method “social contacts with colleagues or classmates at a distance” more than any other coping strategies. Therefore, our participants have been rather deprived of JDCS model’s social support dimension in their occupational environment because social contacts at a distance do not seem to be effective social resources to help the individual efficiently manage the job demands, and consequently to impact job satisfaction positively.

Although we proceeded from JDCS model in the current study, it may be interesting to consider some other perspectives and also the participants’ cultural background to make some explanatory speculations for our minor findings. Below, we discuss the positive impact of two of the most effective coping strategies on telework satisfaction from various perspectives.

### “Make Myself Busy With My Working Day Because It Makes Me Feel Useful”

This pattern of making oneself busy has been also observed in the study conducted by F. Tavares et al. (2021), who found that 39.3% of their respondents indicated that their involvement in working from home had helped them temporarily forget the challenges and worries experienced in the country and in the world. One explanation for why this coping method enhances job satisfaction may be the effect of meaning-making inherent in one’s occupation. According to Friedrich Nietzsche, “He, who has a why to live for, can bear with almost any how.” This reminds us of Weick’s sensemaking theory in industrial and organizational psychology as well. Sensemaking has been defined as the human endeavor to understand ambiguous and complex situations and find meaning in them (Akbari-Zardkhaneh, Fatollahi, & Zandi, 2018). Research has shown that sensemaking helps organizational staff perceive lower ambiguity and higher job satisfaction (Akbari-Zardkhaneh, Zandi, & Qorbani-Vanajemi, 2018).

Furthermore, here, a state like a flow experience is witnessed. Proposed by Csikszentmihalyi (1997), flow experience refers a person’s immersion in a given task, where involvement in that activity is so strong that nothing else seems to matter at the time. In other words, it is a psychological state of maximum optimism and satisfaction, experienced during an activity. While experiencing flow, people maintain control of their own experiences and tend to face challenges; their attention and energies are employed solely to carry out the task. Maeran and Cangiano (2013) showed that flow is considered as a strong predictor of job satisfaction. Chu and Lee (2012) also demonstrated that flow experience positively affects job performance. To experience higher job satisfaction through a flow state, it is necessary for the person to be fully involved in pursuing the activity, which should be simultaneously enjoyable and meaningful for the person. In our research, those participants who report achieving a state similar to flow while teleworking experienced better job satisfaction as well.

In addition, having a clearer idea of what is happening in a culture, that is, wearing “cultural glasses,” may help here. How individuals look at the world is partly founded by their socialization in the culture in which they were brought up (Ahmadi & Zandi, 2021; Ahmadi et al., 2021). Research shows that cultural elements play a great role in adapting to the challenges caused by COVID-19 pandemic (Ahmadi, Cetrez, et al., 2022; Tubadji, 2020). In the current study, the high share of our sample of Iranian academics using the coping strategy “Make myself busy with my working day because it makes me feel useful” and its positive correlation with satisfaction with teleworking can be somehow explained by cultural aspects as well. Looking at this behavioral pattern from a cultural point of view, we may see that the works of some famous Persian poets, whom are loved and read by the Iranian people as mentioned earlier in this article, convey similar messages. For instance, Rumi composed the following (cited in Nicholson, 2007):

“Though Zuleikha shut the doors on every side,

still Joseph gained return (to safety) by bestirring himself.

Lock and door opened, and the way (out) appeared,

when Joseph put trust in God, he escaped.

Though the world hath no visible crevice (means of exit),

(yet) one must run (to and fro) recklessly, like Joseph”.

Also, Hadi Hadavi mentions:

“It is human pride to work

Except for work, one cannot be proud

The product of human life is work

The fruits of life must not be spoiled”

Or in a poem, written by Muhammad Iqbal, a contemporary Persian poet, we read:

“a lively wave loped quickly and said:

I exist if I go, if I don’t go I do not exist”

### “Think About What I Can Do Rather Than What I Can’t Do”

This pattern of thinking about what one can do rather than what one cannot do may be reminiscent of the importance of maintaining hope and doing everything possible to accomplish tasks and experience satisfaction. Research shows that workers who have great levels of self-efficacy and optimism tend to do well in controlling stress and challenges of work since they believe that they are capable of coping with work stressors and challenges (Rubino et al., 2012). Individuals’ self-efficacy, in turn, is perhaps strengthened when they see positive effects of their actions (what they can do).

Moreover, a pattern of positivity and positive thinking orientation (seeing the glass as half full) is somehow observed here. Several studies (Ahmadi et al., 2018; Khodayarifard et al., 2016, 2017, 2021) have explained the phenomenon of positive thinking in Iranian-Islamic culture. As Naseem and Khalid (2010, p. 42) maintained,

positive thinking is looking at the brighter side of situations. It makes a person constructive and creative. The authors explain (ibid.) positive thinking is related with positive emotions and other constructs such as optimism, hope, joy, and wellbeing. McGrath (2004) defined positive thinking as a generic term referring to an overall attitude that is reflected in thinking, behavior, feeling and speaking. Positive thinking is a mental attitude that admits into the mind; thoughts, words and images that are conducive to growth, expansion, and success.

One possible reason for the emergence of this behavioral pattern may be the cultural teachings. There are many poems and aphorisms in this regard in Iranian culture and literature, some of which have been turned into proverbs used in Iranian daily conversations. For example, in a popular poem by Saadi, a well-known Iranian poet, we read:

“Going down the path of a wasteland is better than sitting idly

Even though I might fail my desire, I would try my best”

Also, Rumi, the great Iranian poet, said:

“If you cannot drink the whole sea water, you should drink as much as thirst”

## Conclusion

Iranian university community members were forced to have their workplace in their homes due to the COVID-19 pandemic. The current survey study among a small sample of academics in Iran, concerning job satisfaction and the challenges of telecommuting at times of the pandemic, provides evidence that a majority of participants are very or somewhat satisfied with teleworking. Results also show that the active coping methods “Make myself busy with my working day because it makes me feel useful,”“I care for my mental and physical health,” and “Think about what I can do rather than what I can’t do” have the greatest positive effect on overall job satisfaction. Attempts were made to explain the results through the dimensions of our theoretical framework, JDCS model. We also provided some reflections based on some other approaches, as well as bringing forth more dynamic aspects of culture. We conclude that our Iranian sample can serve as a good example of how academics may control the occupational challenges caused by a worldwide crisis.

This study may be a small attempt to identify and elucidate the role of coping methods and their possible cultural traces in job satisfaction and facing the challenges of working from home during the COVID-19 crisis. The main strength of our research is that, to our knowledge, it is the first study of its kind to investigate job satisfaction in an Iranian university setting in the midst of pandemic concerns. Our study may aid in enriching the knowledge on this topic by discovering how the Iranian university community involved in our study feel about teleworking and tele-education experience, and shedding light on the ways in which they try to deal with the challenges they face. In conducting this study, it is hoped to contribute to an increase in literature in the teleworking field and to allow academic administration officials in Iran to plan evidence-based, culturally tailored teleworking strategies to optimize resources and costs and to improve university operations without harming the quality of life and job satisfaction of faculty/staff members and students during enforced home-working under lockdown.

The major limitations of this study consider the research design, very small sample size, bias in the sample, and the sampling strategy. Generalization of the present findings to different settings and populations should not be done. More comparative national and international studies are needed to support these findings. The preponderance of female, younger, single, and student respondents in the study should be taken into consideration. Another limitation is that the current research employed a cross-sectional design. A longitudinal study would allow us to assess the long-term maintenance of teleworking satisfaction and the coping strategies employed by participants.

### Directions for Future Research

Identifying the factors contributing to workplace health and wellbeing during and beyond COVID-19 is an important and urgent issue in public health today. Conducting similar research in other organizational contexts in Iran (other professional groups who have been forced to telework during the COVID-19 restrictions) and comparing the results with those of the current study may generate valuable knowledge regarding the extent of the variance/invariance of patterns found in this regard. Moreover, exploring the lived experiences of the satisfied and dissatisfied Iranian academics who have been teleworking from home during the COVID-19 outbreak would be a highly relevant subject for phenomenological and phenomenographic qualitative research and inquiries. Additionally, future research could replicate the present study, utilizing a longitudinal research design to examine the long-term consequences of the ongoing pandemic on job characteristics, and to scrutinize the maintenance and persistence of the coping strategies used by academics as the crisis continues to progress. Studies shedding light on the opportunities and challenges of telework in academia will also generate important contributions to the field.

### Practical Implications and Policy Recommendations

- Iranian academics and students are recommended to be active, focus on possibilities, and maintain hope and positivity during working from home and living through a global pandemic. How telework is experienced in academia may be partly dependent on one’s perspective and coping orientation.
- We recommend that Iranian work psychologists, career counselors, and telehealth clinicians and practitioners apply the present results when giving advice to university employees who have problems adjusting to the teleworking restrictions. Human factors and ergonomics principles are also essential issues to be addressed when planning occupational interventions for remote work.
- We encourage higher education and academic administration officials in Iran to provide evidence-based self-study materials and educational classes for academics to inform them of the best science-based ways to maintain their educational well-being and face the obstacles caused by teleworking from home during the pandemic. We also recommend that they provide a space with adequate equipment for staff and students to work/study at home.
- College academic counselors, who provide counseling services for Iranian students and university population, may also benefit from such findings. We suggest that they facilitate online psychology workshops to educate students in how to practice and test effective coping strategies upon encountering difficulties during study-/work-at-home periods.
- The employers are recommended to use the findings of the scientific research in the field of industrial and organizational psychology along with following the workplace hazard controls for COVID-19_ the application of occupational safety and health methodologies for hazard controls to the prevention of COVID-19_ to help the employees maintain health and work-life balance. Even after the end of the COVID-19 pandemic, some public sector and private sector companies may decide to continue to adopt a working from home order in their organizational policies, thereby reducing the number of workers’ daily commuting trips and, consequently, saving on time and costs.
- The Ministry of Science, Research and Technology (Iran) could plan a partial regime of distance education in theoretical courses for the post-pandemic world, the goal being to reduce daily commuting and air pollution. Higher education authorities are also recommended to integrate the employee experiences and behavior in times of past and present crises and also scholars’ views into the future crisis response plans.
- We recommend that academicians, planners and policymakers in Iran allocate more funds and facilities to researchers in the field, so as to facilitate high-quality research on teleworking, flexible working, job quality, COVID-19, coping, psychological capital, and wellbeing at work. Policies also need to be developed concerning external circumstances and factors that are likely to facilitate or hamper the teleworking experience. Research on remote, hybrid, and on-site work deserves more financial support.

## Appendix

Reprinted from *Work*, vol. 71, Ahmadi et al., “Job satisfaction and challenges of working from home during the COVID-19 pandemic: A study in a Swedish academic setting,” pp. 357-370, Copyright (2022), with permission from IOS Press. The publication is available at IOS Press through http://dx.doi.org/10.3233/WOR-210442

**Coping with COVID-19**

We want to ask you if you can take part in a study on the coping methods in relation to the COVID-19.

### Purpose

The following questions relate to the way you have dealt with the situation during the COVID-19 which was declared a pandemic by the World Health Organization (WHO). Countries have begun to take precautions to prevent the spread of the virus, and these precautions have affected individuals and society on all levels. There are many ways to deal with problems. Of course, different people deal with their problems in different ways, and we are interested in how you have dealt with the situation, especially the ones related to your emotions such as worries, fear, and loneliness, which was caused by the spread of the virus in your country and the world. So in this questionnaire, wherever we mention “situation,” we refer to the situation caused by the spreading of the COVID-19, which is characterized by a lack of physical contact with people in general, working at a distance from your regular office, an extreme concern of yours, and your family members’ health, concerns about the economic situation for you personally and for others.

The questions deal with your experiences and shouldn’t result in any uncomfortable situation or risks for you. If you need any consulting due to the question, contact us, and we will provide you with professional support. Your answers to the survey will be saved at the repository of University of Gävle, which is password protected. Only the researchers have access to the material, which is preserved for 10 years. We follow the GDPR directives (EU 2016/679). We will present the results of the study on group and thematic levels only, in scientific publications.

### Consent

1. I have been informed about the study and I give consent to participate in this study:
Yes □ 0 No □ 1
2. I give consent that my answers are handled as described in the information letter:
Yes □ 0 No □ 1
3. In general, would you say your health is:
Poor □ 1 Fair □ 2 Good □ 3 Very good □ 4 Excellent □ 5

### Occupational Health

4. Do you work more than you are contracted each week after you started to work at home as a result of the COVID-19 situation?
No □ 0 Yes □ 1
5. How satisfied are you with your current work from home arrangement?
□ 1 Very dissatisfied
□ 2 Somewhat dissatisfied
□ 3 Neither satisfied nor dissatisfied
□ 4 Somewhat satisfied
□ 5 Very satisfied
6. If you have challenges to work from home, how do you cope with them?

**Table:**

	Never	Seldom	Sometimes	Often	Always
A. I have social contact with my colleagues/classmates through distance tools and other social media	□ 1	□ 2	□ 3	□ 4	□ 5
B. I have social contact with my family and friends through distance tools and social media	□ 1	□ 2	□ 3	□ 4	□ 5
C. I think about what I can do, rather than what I can’t	□ 1	□ 2	□ 3	□ 4	□ 5
D. I make myself busy with my working day because it makes me feel useful	□ 1	□ 2	□ 3	□ 4	□ 5
E. I read/collect information from Public health authorities in my country or World Health Organization and keep myself update with public health news	□ 1	□ 2	□ 3	□ 4	□ 5
F. I trust state or local health authorities in my country	□ 1	□ 2	□ 3	□ 4	□ 5
G. I give myself a news time limit for each day	□ 1	□ 2	□ 3	□ 4	□ 5
H. I avoid recommendations that are not from public health authorities in my county or from World Health Organization.	□ 1	□ 2	□ 3	□ 4	□ 5
I. I care for my mental and physical health	□ 1	□ 2	□ 3	□ 4	□ 5
J. I try offering kindness and support to the people around me	□ 1	□ 2	□ 3	□ 4	□ 5
K. I make sure to have access to medical resources and the health services if I need to seek health care	□ 1	□ 2	□ 3	□ 4	□ 5
L. I believe we are all in this together, and with solidarity we can find the best solutions for handling COVID-19	□ 1	□ 2	□ 3	□ 4	□ 5

Background questions
7. What is your current work/student status?
1 □□ Full-time employment
2 □□ Part-time employment
3 □□ Campus student
4 □□ Distance learning student
8. Which year were you born? …….
9. What is your gender?
1 □□ male
2 □□ female
3 □□ None of the above
10. What is your highest education?
1 □ Lower than elementary school
2 □ Elementary School or equivalent
3 □ Gymnasium or equivalent
4 □ University or equivalent
11. What is your current civil status?
1 □ Married
2 □ Divorced
3 □ Engaged
4 □ Widowed
5 □ Single
6 □ Other (please briefly specify)………………
12. Do you have children?
□ Yes
□ No
13. What characterizes the place you live?
□ Capital
□ Medium-large city, not capital
□ Small city/town close to a large city
□ Small city/town far from a large city
14. What is your country of birth? (Please write)………………………………………
15. Country of residence: (Please write) ……………………………..
